# Prediction of river dissolved oxygen (DO) based on multi-source data and various machine learning coupling models

**DOI:** 10.1371/journal.pone.0319256

**Published:** 2025-03-04

**Authors:** Yubo Zhao, Mo Chen

**Affiliations:** 1 College of Heilongjiang rive and lake chief, Heilongjiang University, Harbin, Heilongjiang Province, China; 2 Institute of Cold Groundwater, Heilongjiang University, Harbin, Heilongjiang Province, China; 3 School of Hydraulic and Electric-power, Heilongjiang University, Harbin, Heilongjiang Province, China; University 20 Aout 1955 skikda, Algeria, ALGERIA

## Abstract

Too low a concentration of dissolved oxygen (DO) in a river can disrupt the ecological balance, while too high a concentration may lead to eutrophication of the water body and threaten the health of the aquatic environment. Therefore, accurate prediction of DO concentration is crucial for water resource protection. In this study, a hybrid machine learning model for river DO prediction, called DWT-KPCA-GWO-XGBoost, is proposed, which combines the discrete wavelet transform (DWT), kernel principal component analysis (KPCA), gray wolf optimization algorithm (GWO), and extreme gradient boosting (XGBoost). Firstly, DWT-db4 was used to denoise the noisy water quality feature data; secondly, the meteorological data were simplified into four principal components by KPCA; finally, the water quality features and meteorological principal components were inputted into the GWO-optimized XGBoost model as features for training and prediction. The prediction performance of the model was comprehensively assessed by comparison with other machine learning models using MAE, MSE, MAPE, NSE, KGE and WI evaluation metrics. The model was tested at three different locations and the results showed that the model outperformed the other models, performing as follows: 0.5925, 0.6482, 6.3322, 0.8523, 0.8902, 0.9403; 0.4933, 0.4325, 6.2351, 0.8952, 0.7928, 0.8632; 0.2912, 0.2001, 4.0523, 0.7823, 0.8425, 0.8463 and the PICP values exceed 95%. The hybrid model demonstrated significant results in predicting dissolved oxygen concentrations for the next 15 days. Compared with other studies, we innovatively improved the prediction accuracy of the model significantly through noise removal and the introduction of multi-source features.

## Introduction

River dissolved oxygen is an important indicator of water quality and ecosystem health, and it is affected by a variety of environmental factors. Insufficient dissolved oxygen can lead to the inhibition of aquatic biological activity or even ecosystem collapse; while too much can cause eutrophication and algal blooms, posing a serious threat to river ecosystems and water quality management [1]. However, traditional prediction models have limited accuracy and applicability because they have difficulty capturing the complex nonlinear relationship between dissolved oxygen and environmental factors. This study aims to combine key water quality parameters with advanced machine learning algorithms to establish a highly accurate dissolved oxygen prediction model to solve the prediction problem and provide a reliable scientific tool for river ecological protection and water quality management [2].

Dissolved oxygen is a key water quality parameter for evaluating the water body rating. There are two main types of dissolved oxygen concentration prediction models: 1. traditional models based on physics, and 2. data-driven models based on artificial intelligence. Traditional prediction models simulate the physical mechanisms in rivers and describe the transfer process of pollutants in water. Physical models can elucidate the hydrodynamic mechanism and show the temporal and spatial trends of pollutants. A number of studies have used physical models to predict water quality and dissolved oxygen. For example, Zehra et al. used the water quality analysis simulation program (WASP) model and the QUAL 2Kw model to systematically analyze the dissolved oxygen and other water quality indicators of the Yamuna River and predict future spatial trends [3]. Zhang et al. used the Mikezl model to simulate the impact of ecological recharge on river hydraulics and discuss the trend of DO under different hydrological conditions. However, the model often requires a large amount of hydrological data during operation, which limits the establishment of models for rivers with fewer data sets [4]. In addition, traditional mechanistic models usually rely on the establishment of hydrodynamic equations, and their internal parameters are complex.

In recent years, with the development of machine learning algorithms, the application of AI-based data-driven models in dissolved oxygen prediction has gradually attracted attention. Compared with models based on physical mechanisms, data-driven models have a simple modeling process and do not require complex mathematical formulas and parameter settings. Faced with multi-factor, non-linear dissolved oxygen sequence data, AI-based data-driven models can capture their characteristics.

Common data-driven models include artificial neural networks (ANN), support vector machines (SVM), random forests (RF), and long short-term memory networks (LSTM), among others. Li, Dashe, et al. constructed a water quality parameter prediction model by combining a discrete hidden Markov model (DHMM) with a K-means clustering method. The model was used to predict the dissolved oxygen saturation of six marine pastures in the Bohai Sea. The DHMM hidden states affecting the target water quality parameters were determined based on the analysis of the correlation between various water quality parameters, which improved the adaptability of the model. The number of hidden states was determined using K-means clustering, and a DHMM-based water quality parameter prediction model was established to predict dissolved oxygen saturation [5]. Huang, Sheng, et al. used neural networks (RNN), long short-term memory networks (LSTM), and gated recurrent units (GRU) to predict in the Dahuihe River Basin using meteorological conditions as input features for prediction. The LSTM and GRU showed excellent prediction capabilities on daily measurements and can effectively predict multiple water quality parameters. It was also discussed that meteorological data can effectively drive deep learning models to predict water quality parameters [6]. Li, Shuguang, et al. established four machine learning models, including support vector regression (SVR), gradient boost (GB), and random forest (RF) to model and predict the minimum, maximum, and dissolved oxygen concentration (DOmin, DOmax, DOmean) at a monitoring station on the American Tualatin River. The results showed that the SVR and MLP models performed similarly to the RF and GB models. Among them, SVR performed best in explaining DOmin, DOmax and DOmean [7]. Wang, Zhaocai, et al. Segmented the original dissolved oxygen data into multiple subsequences using VMD for training, and each subsequence was predicted by GRU. The final prediction result was the sum of the predicted values of each subsequence. The DM test results showed that at a significance level of 1%, the prediction accuracy of this model was significantly higher than that of other benchmark models [8]. Singh, Rosysmita Bikram, et al. utilised the hidden droplet optimisation algorithm HDTO to adjust the parameters and construct a model for the deep autoregressive network model DeepAR. The water quality indicators of the Mahatma Nadi River Basin were predicted, and the results showed that dissolved oxygen will maintain a dynamic balance in the future [9]. Tian, Yuqing, et al. developed a model framework that integrates features into water quality prediction using a spatio-temporal graph convolutional network (STGCN), and found that potential pollution areas are mainly concentrated in the southwestern and middle and lower reaches of the Yangtze River Basin. The efficacy of the developed framework in predicting potential pollution areas in large river basins was confirmed by the study’s findings [10].zhu et al. used water temperature, pH, ammonia and conductivity as eigenvalues of the model input to the ELM and multilayer perceptron in predicting day-by-day DO concentrations, and the results showed that their model exhibited poor robustness in the face of different hydrological conditions[11]. Achite, Mohammed, et al., 2023. “Modelling the sodium adsorption ratio (SAR) in irrigation water using hybrid swarm intelligence-based neural networks provides agricultural practitioners and policymakers with an effective tool for sustainable agriculture in semi-arid regions by screening key water quality parameters and optimising modelling methods.” [12]

However, previous studies have predominantly concentrated on adjusting model parameters and constructing frameworks, frequently overlooking the impact of meteorological factors and the potential noise in features on prediction accuracy. To address these issues, we established a dissolved oxygen prediction model that fuses multiple data sources and multiple machine learning methods, called DWT-KCPA-GWO-XGBoost. A novel integration of meteorological elements was introduced into the dissolved oxygen prediction model, leading to the effective denoising of features that contained significant amounts of noise. This approach resulted in enhanced prediction accuracy of river dissolved oxygen concentration.

## Methods

This section demonstrates the methods used to construct the dissolved oxygen (DO) prediction model and describes in detail the implementation of these methods to provide theoretical support for the model. In order to test its robust shape, we arranged the model in three locations with different latitude and longitude.

### The feature mapping of nonlinearly-coupled variables

Firstly, the dataset $X_{nonlinear}^{'}$ , which is of non-linear correlation with Y, is entered into the kernel principal component analysis to take a nonlinear spatial mapping, and the new data $X_{nonlinear}^{'}$ can be obtained, which is the feature map in linear space of $X_{nonlinear}$, as is shown [13]:

Initially, the proposed methodology has to choose the kernel function and compute the kernel matrix K. Here, a common Gaussian RBF is selected as:


$$k(x_{1},x_{2})=\exp(-\frac{||x_{1}-x_{2}|{|}^{2}}{\sigma^{2}})$$ (1)

Thus, if the data in the feature space do not satisfy the centering condition, the kernel matrix is normalized according to the formula:


$${\bar{k}}_{ij}=k_{ij}-\frac{2}{n_{1}}\overset{n_{1}}{\underset{m_{1}=1}{\sum }}k_{m_{1j}}+\frac{1}{{n_{1}}^{2}}\overset{n_{1}}{\underset{m_{1},m_{2}=1}{\sum }}k_{m_{1}m_{2}}$$ (2)

Where, $n_{1}$ is the number of variables in the set $X_{nonlinear}$.

Now the eigenvalues ${\text{λ}}_{1}$, ${\text{λ}}_{2}$, ... , ${\text{λ}}_{n}$ and eigenvectors $v_{1}$, $v_{2}$, ... , $v_{n}$ of $\overset{-}{k}$ can be calculated. The latter can be sorted in descending order of the corresponding eigenvalues and then be normalized via the Gram–Schmidt orthogonal method to get new feature vectors as ${\text{a}}_{1}$, ${\text{a}}_{2}$, ... , ${\text{a}}_{n}$. On the consideration of the cumulative contribution rate $B_{1}$, $B_{2}$, ... , $B_{n}$ of the eigenvalues, the principal components ${\text{a}}_{1}$, ${\text{a}}_{2}$, ... , ${\text{a}}_{t}$ are extracted if $B_{t}$ ≥  *p*, where, *p* is a given extraction efficiency.

After that, the mapped variables $X_{nonlinear}^{'}$ are calculated by the modified kernel matrix $\overset{-}{k}$.


$$X_{nonlinear}^{'}=\;\overset{-}{k}a$$ (3)

which a = (${\text{a}}_{1}$, ${\text{a}}_{2}$, ... , ${\text{a}}_{t}$).

### XGBoost

XGBoost is the abbreviation for extreme gradient boosting, and is a supervised EL algorithm that represents a very large gradient boosting algorithm that places inside it a regularization term to yield high-accuracy models for classification, regression, and ranking in multicore and distributed setups[14,15,16,17]. Given a data set with *n* instances and m attributes $X={\left\{x_{i}\right\}}_{i=1}^{n}$, $x_{i}\in R^{m}$, and labels $y={\left\{y_{i}\right\}}_{i=1}^{n}$, $y_{i}\in \{\omega_{\forall j\in (1,2,\ldots ,C)}\}$, where, $\omega_{j}$ symbolizes the $j^{\text{th}}$ class among *C* total classes, a set of DT employing *K* additive functions for output prediction can be created as:


$${\hat{y}}_{i}=\sum_{k=1}^{K}f_{k}(x_{i}),f_{k}\in F$$ (4)

Where, $F=\{f(x)=w_{q}(x)\}(q:R^{m}\to T,w\in R^{T})$ represents the realm of CART. In this context, *q* and *T* symbolize the tree’s structure and leaf count, with each tree $f_{k}$ representing a separate *q* and leaf weights *w*. In a specific case, decision-making rules from *q* ‘s tree structure will categorize it into leaves, and the ultimate forecast will be made by aggregating the scores from *w* ‘s respective leaves. Subsequently, the ensuing regularization term aids in understanding the functions employed in the ensemble model:


$$l=\sum_{i=1}^{n}l({\hat{y}}_{i},y_{i})+\sum_{k=1}^{K}\Omega (f),where\Omega (f_{k})=\xi T+\frac{1}{2}\xi ∥w{∥}^{2}$$ (5)

Where, *l* is a differentiable convex loss function that measures the difference between the prediction ${\hat{y}}_{i}$ and the target $y_{i}$ and the second term Ω(f) describes the complexity of tree $f_{k}$, where, ξT and $\xi ∥w{∥}^{2}$ penalize each tree leaf involved in addition and extreme weights, respectively[18].

Regrettably, the Equation encompasses parametric functions, making it impractical to optimize it through conventional Euclidean space optimization techniques. Nonetheless, the model’s additive training approach allows us to articulate the objective function for the present iteration *t* based on the prior iteration t−1 ’s prediction, modified by the latest tree $f_{t}$:


$$l^{\left(t\right)}=\sum_{i=1}^{n}l({\hat{y}}_{i}^{\left(t-1\right)}+f_{t}(x_{i}),y_{i})+\Omega (f_{t})$$ (6)

By taking the Taylor expansion of Eq. (6) to the first- and second-order gradients based on the loss function, we can obtain the following simplified objective function:


$$l^{\left(t\right)}≅\sum_{i=1}^{n}\left[g_{i}f_{t}(x_{i})+\frac{1}{2}h_{i}f_{t}^{2}(x_{i})\right]+\Omega (f_{t})$$ (7)

Where, $g_{i}=\partial_{{\hat{y}}^{\left(t-1\right)}}l(y_{i},{\hat{y}}^{\left(t-1\right)})$ and $h_{i}=\partial_{{\hat{y}}^{\left(t-1\right)}}^{2}l(y_{i},{\hat{y}}^{\left(t-1\right)})$.

A DT predicts constant values within a leaf. Thus, tree $f_{k}(x)$ can be represented by $w_{q}(x)$, where, *w* is the score vector for each leaf and q(x) maps instance *x* to a leaf. By expanding the second term in Eq. (7), a sum over the tree leaves can be obtained, and the regularization term becomes:


$$l^{\left(t\right)}≅\sum_{j=1}^{T}\left[G_{j}w_{i}+\frac{1}{2}(H_{j}+\xi )w_{j}^{2}\right]+\lambda T,where{\text{G}}_{j}=\sum_{j\in I_{j}}g_{i},H_{j}=\sum_{j\in I_{j}}h_{i}$$ (8)

Where, $I_{j}=\{i|q(x_{i})=j\}$ is the instance at leaf *j*.

For a fixed structure tree, the objective function can be minimized as $\partial l^{\left(t\right)}/\partial w_{j}=G_{j}+(H_{j}+\lambda )w_{j}=0$, and the best weight of leaf *j* can be obtained by:


$$w^{*}=-\frac{G_{j}}{H_{j}+\xi }$$ (9)

By substituting this formula into Eq. (8), the objective function for finding the best tree structure then becomes:


$$l^{\left(t\right)}≅-\frac{1}{2}\sum_{j=1}^{T}\frac{G_{j}^{2}}{H_{j}+\xi }+\xi T$$ (10)

In XGBoost with a DART booster, suppose *k* trees were dropped from the algorithm during the *m* -th training round. Let $D=\sum_{k\in K}F_{k}$ be the leaf scores of the dropped trees and $F_{m}=\eta {\tilde{F}}_{m}$ be the leaf scores of a new tree; then, the objective function form in Eq. (6) can be reformed as:


$$l^{\left(t\right)}=\sum_{i=1}^{n}l({\hat{y}}_{i}^{\left(m-1\right)}-D_{i}+{\tilde{F}}_{m},y_{i})+\Omega ({\tilde{F}}_{m})$$ (11)

Where, *D* and $F_{m}$ are overshooting parameters that are supposed to be normalized in actual applications, XGBoost has tree and forest-based normalization, too.

For XGBoost with the linear booster, the form of the objective function is given as:


$$\begin{array}{c} l^{\left(t\right)}=\frac{1}{n}\sum_{i=1}^{n}l({\hat{y}}^{\left(t-1\right)},y_{i})+\Omega (\omega ,b)\\ =\frac{1}{n}\sum_{i=1}^{n}l({\hat{y}}^{\left(t-1\right)},y_{i})+\frac{1}{2}\lambda ∥\omega {∥}^{2}+\frac{1}{2}\lambda_{b}b^{2}+a∥\omega ∥{\;}_{1} \end{array}$$ (12)

Where, y=ωx+b, $\omega =(\omega_{1},\omega_{2},\ldots ,\omega_{d})$ is a linear model, *d* is the dimension of features, *λ* is the $l_{2}$ regularization term based on *ω*, $\lambda_{b}$ is the $l_{2}$ regularization term based on the offset coefficient *b*, and *a* is the $l_{1}$ regularization term based on *ω*.

### Gray wolf optimization (GWO)

The GWO algorithm operates on the natural ranking and hunting patterns of gray wolves. This method is applicable for problem-solving and optimization. Addressing the optimization issue involves four distinct methods: ranking gray wolves, tracking them, encircling, and exterminating their prey [19].

Initially, an arbitrary starting population was created within the decision-making framework. Subsequently, based on a top-to-bottom natural hierarchy, the gray Wolf population was divided into four distinct categories: *α*, *β*, *δ*, *ω*. Among them, *ω* is the leader of the grey wolves and represents the leading grey wolf other than the remaining three grey wolves [20].


$$Q(t+1)=Q_{p}(t)-(2a\cdot \rho_{1}-a)(2\rho_{2}\cdot Q_{p}(t)-Q(t))$$ (13)

a=2−2⋅it/MAX−IT (14)

Where, *t* stands for the number of iterations, $X_{p}(t)$ presents position vector of the prey, X(t) is position vector of the current gray wolf, X(t+1) represents the position of gray wolf in the next iteration. a can be calculated by Eq. (14) and it’s value decreases linearly from 2 to 0. Where it stands for the current number of iterations, and MAX_IT represents the maximum number of iterations. $\rho_{1}$ and $\rho_{2}$ are both the random numbers in [0,1].

Three of the best gray wolves represented the best population of GWO, but by no means were they at risk of extinction. Because of this, one assumes that they are able to track prey with a certain degree of expertise. Simulate the process of the hunting of gray wolves. The next step would involve the updating of equations in GWO to mark the position of other wolves.

### DWT-db4

Discrete Wavelet Transform (DWT) uses the basic wavelet with scale discretized. In comparison with Fourier Transform, the original signal is downward decomposed. At each decomposition layer, there are high-scale low-frequency signals (which are included in the following decomposition response, other high and low-frequency signals can be ignored) and low-scale high-frequency signals, named as Approximation Coefficients and Detail Coefficients, respectively. Since the data used in this study is sequential, DWT is adopted. The corresponding expression for DWT is as follows [21,22]:


$$C_{a}=\sum_{n=0}^{\infty }f\left(n\right)\phi_{j,k}\left(n\right)$$ (15)

$$\phi_{j,k}\left(n\right)=2^{\left(-j/2\right)}\phi \left(2^{-j}n-k\right)$$ (16)

Where, $f\left(n\right)$ is the original signal. $\phi_{j,k}\left(n\right)$ is Scaling Function; j controls the dilation or translation;k denotes the position of the wavelet function.


$$C_{d}=\sum_{n=0}^{\infty }f\left(n\right)\varphi_{j,k}\left(n\right)$$ (17)

$$\varphi_{j,k}\left(n\right)=2^{\left(-j\right)}\phi \left(2^{-j}n-k\right)$$ (18)

Where, $\varphi_{j,k}\left(n\right)$ is Wavelet Function.


$$DWT_{f}\left(m,n\right)=\frac{1}{\sqrt{a^{m}}}\int_{-\infty }^{+\infty }f(t)\psi^{*}(\alpha_{0}^{-m}t-n\tau_{0})dt$$ (19)

Where, $\alpha_{0}$ is the scale parameter; $\tau_{0}$ is the translation parameter; m,n are the scaling and translation parameters, respectively; $\psi^{*}$ is the complex conjugate function; f(t) is the time series.

A total of eight wavelet basis functions were selected for comparison, and the most suitable noise reduction method was determined, with the conductivity of the S1 station serving as the criterion. A higher Retained Energy Ratio value indicates that the main information in the signal is better retained and the noise component is more effectively removed; a higher Sparsity value indicates that the signal is compressed more cleanly and is suitable for further feature extraction or compression analysis; a smaller Mean Squared Error value indicates that the denoised signal is closer to the original signal and the denoising effect is better. As demonstrated in Table 1. When db-4 is used for noise reduction, sparsity, retained energy ratio and mean squared error (Mae) are 93.62, 99.94 and 4.779 respectively, which are better than those of other wavelet-based functions.

**Table 1:** Comparison of the eight wavelet basis functions for signal processing.

Types	Sparsity (%)	Retained energy ratio (%)	Mae
Db-4	93.62	99.94	4.779
Haar-4	87.25	98.52	5.963
Sym-4	70.63	93.54	7015
Coif-4	89.36	99.33	5.212
Bior-4	77.56	95.85	7.052
Rbio-4	80.62	97.21	6.596
Dmey-4	86.26	98.23	6.125
Fk-4	90.36	99.56	4.962

### Performance metrics

We have used several indicators to comprehensively assess the performance of the model. The formula for the statistical indicator is as follows:


$$MSE=\frac{\sum {\left({\text{y}}_{i}-{\hat{y}}_{i}\right)}^{2}}{n}$$ (20)

$$MAE=\frac{1}{n}\sum_{i=1}^{n}\left|{\text{y}}_{i}-{\hat{y}}_{i}\right|$$ (21)

$$MAPE=\frac{1}{n}\sum_{i=1}^{n}\left|\frac{{\text{y}}_{i}-{\hat{y}}_{i}}{{\text{y}}_{i}}\right|*100\%$$ (22)

$$NSE=1-\frac{\sum_{i=1}^{n}{\left(y_{i}-{\hat{y}}_{i}\right)}^{2}}{\sum_{i=1}^{n}{\left(y_{i}-{\bar{y}}_{i}\right)}^{2}}$$ (23)

$$KGE=1-\sqrt{{\left(r-1\right)}^{2}+{\left(\beta -1\right)}^{2}+{\left(\gamma -1\right)}^{2}}$$ (24)

$$WI=1-\frac{\sum {\left(y_{i}-{\hat{y}}_{i}\right)}^{2}}{\sum {\left({\left|{\hat{y}}_{i}-{\bar{y}}_{i}\right|}^{2}+\left|y_{i}-{\bar{y}}_{i}\right|\right)}^{2}}$$ (25)

where, $y_{i}$ is the actual value; ${\hat{y}}_{i}$ is the predicted value; ${\bar{y}}_{i}$ is the mean actual value; *n* is the number of observations in the dataset; *r* is the correlation coefficient; *n* is the ratio of the predicted mean to the actual mean.

Mean Squared Error (MSE) represents the mean of the sum of the squares of the difference between the predicted value and the actual value of the model. MSE is more sensitive to values with large prediction errors [23]. Unlike MSE, Mean Absolute Error (MAE) calculates the mean of the squares of the errors between the predicted and actual values [24]. Mean Absolute Percentage Error (MAPE) measures the mean of the absolute values of the errors between the predicted and actual values as a percentage of the actual values [25]. Nash-Sutcliffe Efficiency (NSE) not only focuses on the absolute value of the model error, but also considers the variability of the data, so it can better evaluate whether the model captures the true variability of the data [26]. Kling-Gupta Efficiency (KGE) is a comprehensive model evaluation index that measures the overall prediction performance of the model. It considers the difference between the predicted value and the actual value and the model’s ability to capture the trend and range of data changes [27]. Willmott’s Index of Agreement (WI) measures the consistency between the predicted value and the measured value [28].

The smaller the MSE, MAE and MAPE values, the closer the model’s predicted results are to the actual values and the higher the model’s accuracy. The closer the NSE and WI values are to 1, the better the predictive power of the model. The KGE comprehensively considers correlation, mean deviation and range deviation, and can evaluate model performance from multiple perspectives. The closer the KGE is to 1, the better the model can fit the trend, mean and range of the actual data[29].

We also set a 95% confidence level. If the Prediction Interval Coverage Probability (picp) is > 95%, it means that the true value is basically within the prediction interval.


$$PICP=\frac{1}{n}(\sum_{i=1}^{n}d_{i})\times 100$$ (26)

Where, if the true value falls within the predicted interval, then $d_{i}$ = 1, otherwise $d_{i}$ = 0.

### Correlation analysis

There is some potential relationship between dissolved oxygen and environmental factors. Therefore, the characteristics that are significantly correlated with dissolved oxygen are identified by the Pearson correlation coefficient as input to the model, and the characteristics with weak correlation are discarded to reduce the complexity of the model.

The Pearson correlation coefficient is used to calculate the correlation coefficient of multiple factors, and factors with correlation coefficients are selected for inclusion in the model.The formula for calculating the Pearson correlation coefficient is as follows:


$$p(x,y)=\frac{\sum_{i=1}^{n}\left(x_{i}-\bar{x})(y_{i}-\bar{y}\right)}{\sqrt{\sum_{i=1}^{n}{\left(x_{i}-\bar{x}\right)}^{2}}\sqrt{{\left(y_{i}-\bar{y}\right)}^{2}}}$$ (27)

Wher, $x_{i}$, $y_{i}$ is the *i* -th element of the two vectors *x* and *y*, respectively; $\bar{x}$, $\bar{y}$ is the mean values of elements in *x* and *y*, respectively.

### DWT-KCPA-GWO-XGBoost

**Fig 1:**
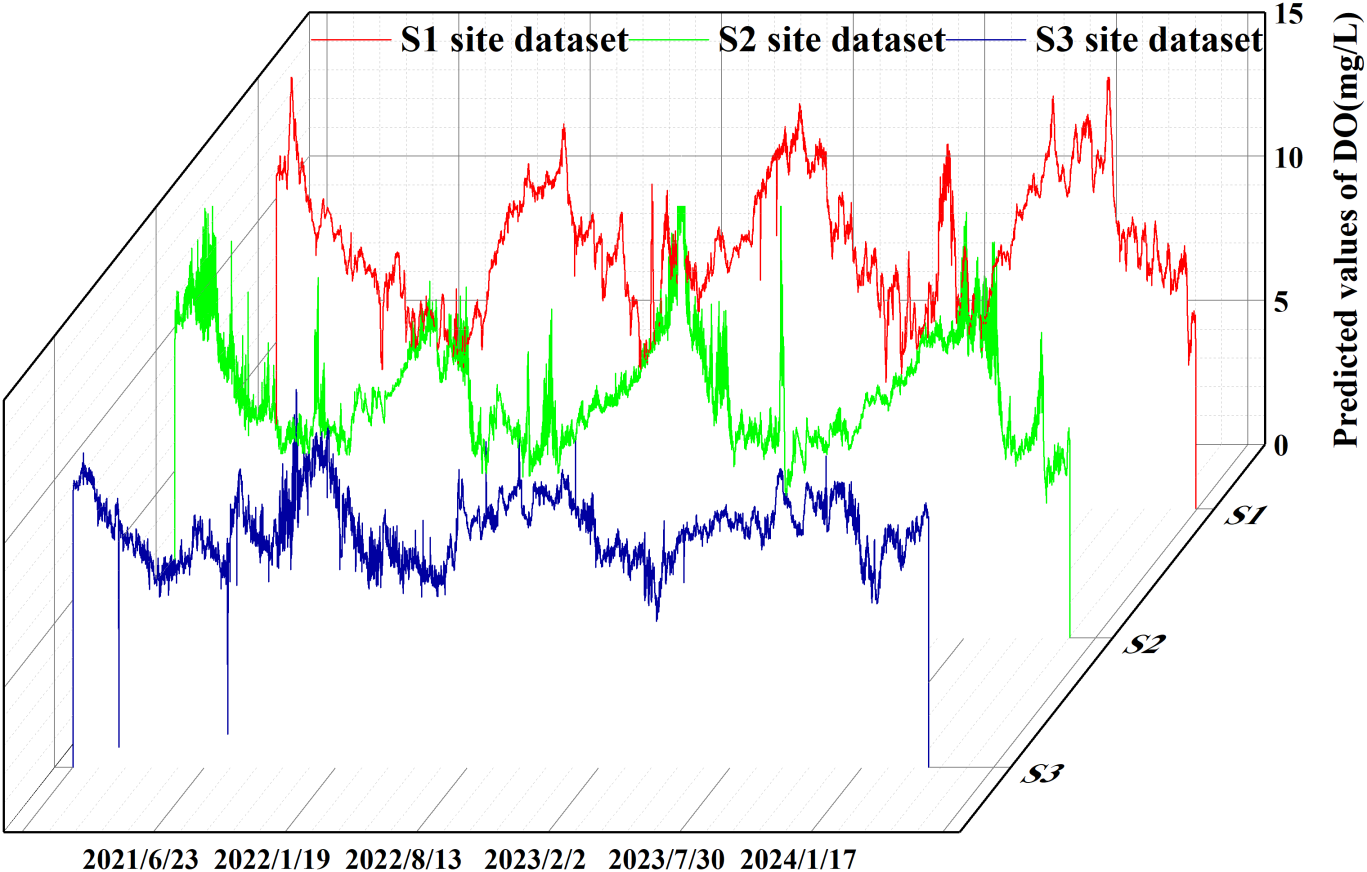
Three station data sets.

**Fig 2:**
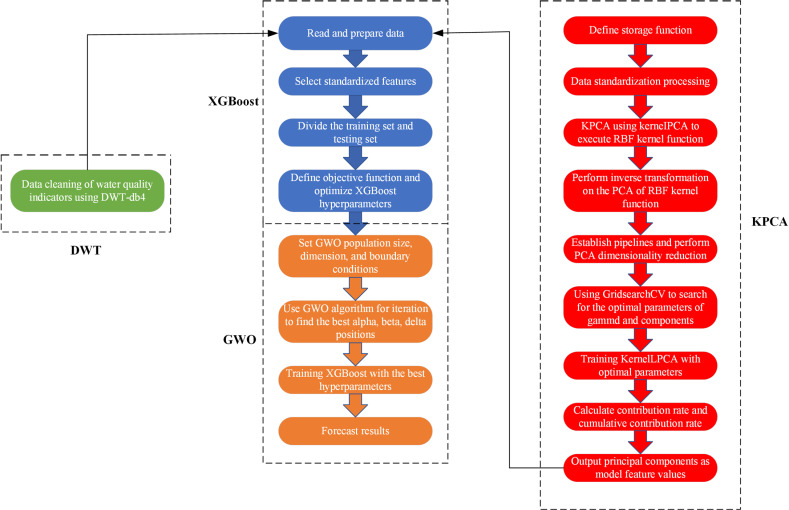
DO prediction framework.

As can be seen in Fig 1, the dissolved oxygen curve is nonlinear and random. We propose a combined model called DWT-KPCA-GWO-XGBoost. First, we found that there is a lot of noise in several water quality parameters, and once they are used as the model’s feature inputs, the noise may reduce the model’s accuracy. The noisy water quality parameters are conductivity and turbidity, so we use discrete wavelet transform to denoise conductivity and turbidity [30]. In addition, this study believes that meteorological factors can affect the change of dissolved oxygen concentration. Therefore, we use meteorological data as the feature values for predicting DO. Considering that too many feature values will complicate the prediction model, we reduce the dimensionality of the eight meteorological factors into four main components, which contain 90% of the data information. Finally, we performed a Pearson correlation analysis on the meteorological principal components, the denoised water quality parameters and other water quality parameters to select suitable features for input into GWO-XGBoost. The specific process is shown in Fig 2.

### Case analysis

We selected water quality and meteorological data from three stations for dissolved oxygen prediction. The station information and data sources are described below.

### Three station locations

The three stations of this study are the confluences of the Yangtze mainstream with the Minjiang, Tuojiang and Hanjiang rivers, which we have named S1, S2 and S3.

he Yangtze River Basin lies between 24° and 36° north latitude and 90° and 123° east longitude and covers an area of about 1.8 million square kilometres. It is the most important river basin in China. From top to bottom, the Yangtze River Basin flows through the Qinghai-Tibet Plateau, the Hengduan Mountains, the Yunnan-Guizhou Plateau, the Sichuan Basin, the Jiangnan Hills and the Middle and Lower Yangtze River Plain.

The Minjiang River is located in the centre of Sichuan Province, between 102°32’ and 104°54’ east longitude and 27°49’ and 33°09’ north latitude. It rises at Langjialing at the southern foot of the Min Mountains in Songpan County, flows through Songpan and Mao Counties, and finally joins the main channel of the Yangtze River at Yibin. It is 1,279 kilometres long and holds one-fifth of the Yangtze’s total water resources. The Minjiang River basin covers nine cities (prefectures) and 45 counties (districts), namely Aba Tibetan and Qiang Autonomous Prefecture, Chengdu City, Ya’an City, Meishan City, Zigong City, Liangshan Yi Autonomous Prefecture, Leshan City, Neijiang City and Yibin City.

Located in the eastern part of the Sichuan Basin, the Tuo River covers an area of approximately 38,600 square kilometres and includes the cities of Deyang, Chengdu, Meishan, Ziyang, Leshan, Neijiang, Zigong, Luzhou and Yibin.

**Fig 3:**
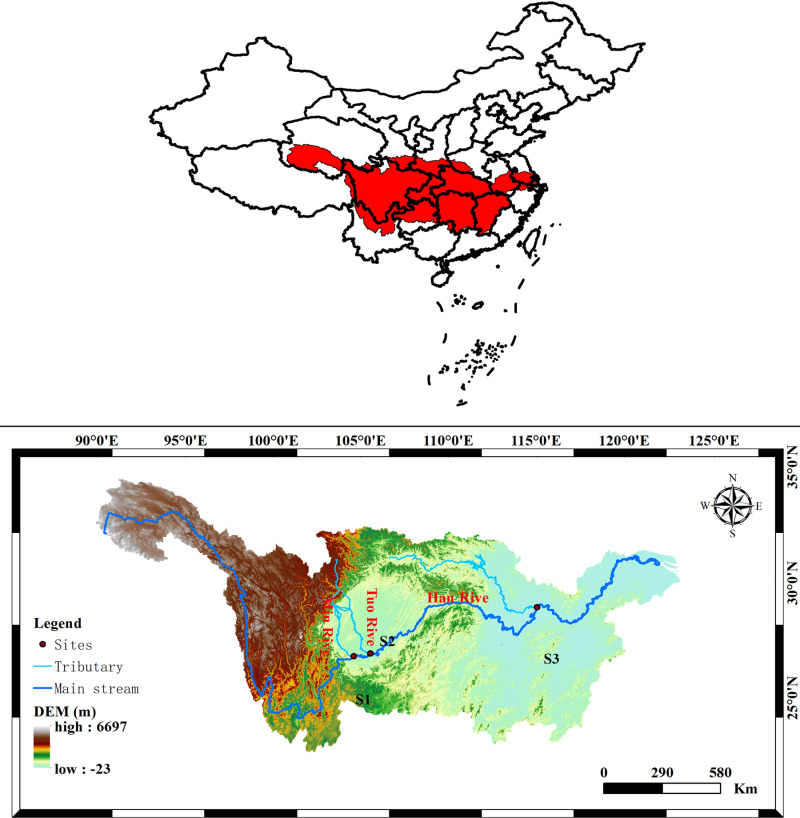
Water quality monitoring stations in the changjiang River.

The Han River is located between 30°05’–34°25’ north latitude and 105°30’–114°00’ east longitude. The mainstream flows through 64 counties and districts in Shaanxi, Henan and Hubei provinces, with a total length of about 1,532 kilometres and a basin area accounting for about 1.59% of the Yangtze River basin. The Han River is the largest tributary of the Yangtze. It is located in a subtropical monsoon climate zone and is a strategic waterway connecting the Yangtze River Economic Belt and the New Silk Road Economic Belt. Fig 3 shows the location of the monitoring stations.

### Three station datasets

Data from China National Environmental Monitoring Centre (CNEMC) and China Meteorological Data Centre (CMD). Period from 1 January 2021 to 30 June 2024. Data are automatically retrieved at 0:00, 4:00, 8:00, 12:00, 16:00 and 20:00. Water quality indicators include water temperature (T), pH, dissolved oxygen (DO), ammonia nitrogen (NH3-N), total phosphorus (TP), total nitrogen (TN), turbidity (TUR) and electrical conductivity (EC); meteorological elements include temperature (T), humidity (%), wind speed (m/s), wind direction (degrees), air pressure (hPa), precipitation (mm) and visibility (km). During the data collection process, data may be missing due to station failures or human factors. To ensure the continuity of the data, the Lagrange interpolation method is used to interpolate the data to obtain a complete time series of water quality data. Detailed data are given in Table 2.

**Table 2:** Data details.

Station		Elements	Group	Missing value		Elements	Group	Missing value
Zong guan (S1)	water quality data	T	6991	/	Meteorological Data	T	6991	30
		PH	6991	/		Humidity	6991	/
		DO	6991	53		Wind speed	6991	/
		NH3-N	6991	62		Wind direction angle	6991	330
		TP	6991	112		Air pressure	6991	123
		TN	6991	140		Hourly precipitation	6991	325
		EC	6991	70		Visibility	6991	/
		TUR	6991	26				
Tuo jiang Bridge (S2)		T	6808	/		T	6808	/
		Ph	6808	/		Humidity	6808	/
		DO	6808	/		Wind speed	6808	163
		NH3-N	6808	35		Wind direction angle	6808	/
		TP	6808	263		Air pressure	6808	/
		TN	6808	136		Hourly precipitation	6808	56
		EC	6808	/		Visibility	6808	/
		TUR	6808	/				
Liang jia gou (S3)		T	6509	/		T	6509	/
		PH	6509	/		Humidity	6509	53
		DO	6509	/		Wind speed	6509	/
		NH3-N	6509	335		Wind direction angle	6509	/
		TP	6509	43		Air pressure	6509	24
		TN	6509	6		Hourly precipitation	6509	/
		EC	6509	/		Visibility	6509	/
		TUR	6509	/				

### Meteorological data reduction dimension

**Fig 4:**
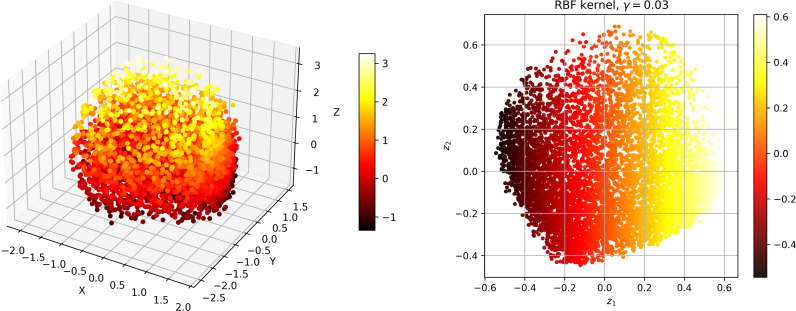
S1 station data visualised after inverse transformation and visualised after dimensionality reduction.

**Fig 5:**
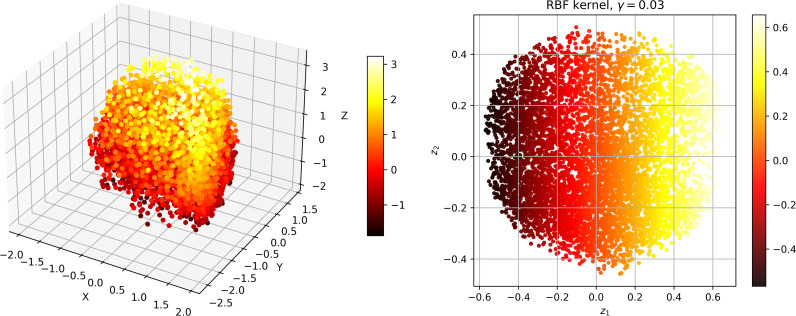
S2 station data visualised after inverse transformation and visualised after dimensionality reduction.

**Fig 6:**
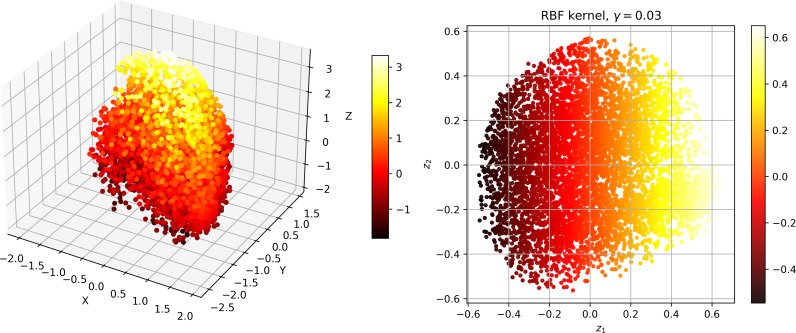
S3 station data visualised after inverse transformation and visualised after dimensionality reduction.

**Fig 7:**
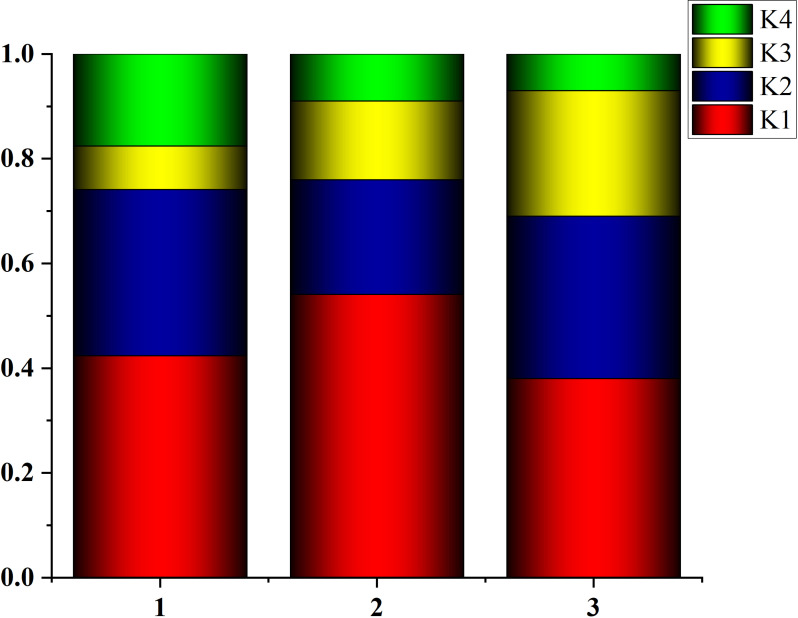
The maximum eigenvalue contribution rates of meteorological principal components at three stations.

To investigate the correlation between dissolved oxygen and meteorological factors, we downscale the meteorological data. The Kernel Principal Component Analysis (KPCA) method is used to map high-dimensional, non-linear and indivisible data into high-dimensional space, and then the dimension is reduced to linear and divisible low-dimensional data by Principal Component Analysis (PCA) [31]. Figs 4–6 show the dimension reduction of the elements, and Fig 7 shows the cumulative contribution rate of the elements.

### Features analysis

**Fig 8:**
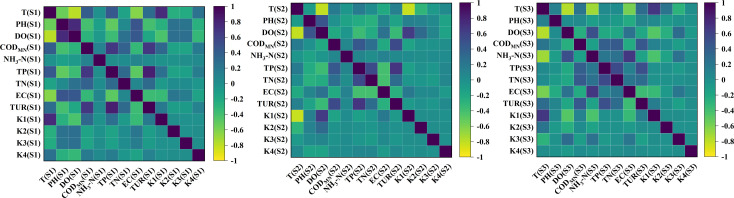
Pearson correlation coefficient matrix heat map.

In DO prediction, changes occurring in the DO concentration tend to follow a trend that is influenced by other water quality factors and meteorological data. Many studies have shown that the ability to include external factors would enhance the model accuracy [32,33]. We used Pearson for feature screening of the model. Fig 8 shows the study of external feature correlation.

The strength of correlation is generally evaluated within a numerical range of 0.8–1.0 being very strong, 0.6–0.8 being strong, 0.4–0.6 being moderate, 0.2–0.4 being very weak or unrelated, and 0.0–0.2 being uncorrelated. Therefore, T, PH, EC, TUR, k1 and K4 were selected as the characteristic values for the prediction model.

### Noise reduction processing

**Fig 9:**
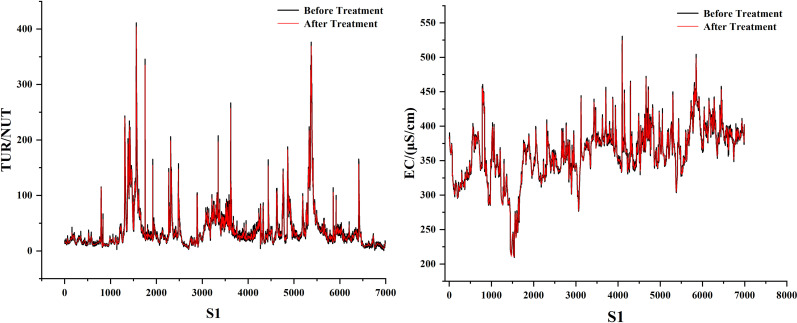
S1 station noise reduction result.

**Fig 10:**
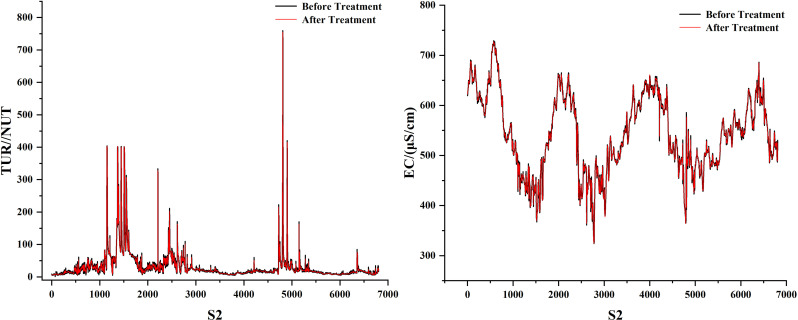
S2 station noise reduction result.

**Fig 11:**
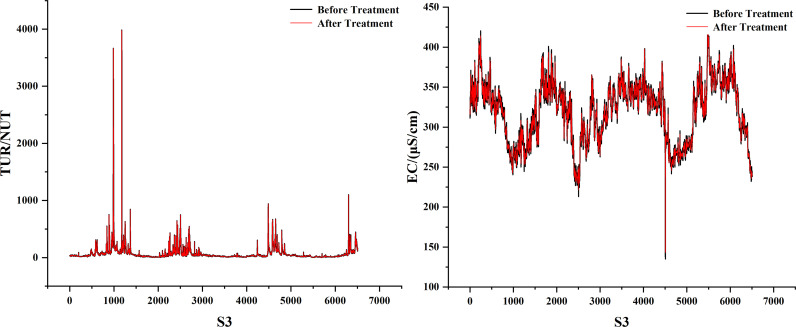
S3 station noise reduction result.

We found that among all the eigenvalues, EC and TUR have many mutated values due to the influence of unknown factors. Therefore, the db-4 function of the discrete wavelet basis is used to denoise them. Figs 9–11 show the denoising visualisation of the three stations.

## Results

### Predicted results

We placed the proposed models at three stations with different hydrological conditions and comprehensively measured the advantages and disadvantages of the models using seven evaluation indicators. Table 2 shows the error analysis of each model at stations S1–S3. We placed the proposed models at three stations with different hydrological conditions and comprehensively measured the advantages and disadvantages of the models using seven evaluation indicators. Table 3 shows the error analysis of each model at stations S1–S3.

**Table 3:** Comparative analysis of DWT-KCPA-GWO-XGBoost with other predictive models.

WQI	Stations	Models	MAE	MSE	MAPE%	NSE	KGE	WI
DO	S1	DWT-KCPA-GWO-XGBoost	0.5925	0.6482	6.3322	0.8523	0.8902	0.9403
		KCPA-GWO-XGBoost	0.6158	0.6852	6.4526	0.8235	0.8632	0.9214
		GWO-XGBoost	0.6629	0.7364	6.5843	0.8150	0.8236	0.9022
		PSO-XGBoost	0.6936	0.8182	6.9362	0.6882	0.7253	0.8021
		XGBoos+ANN	0.8345	1.0967	7.8276	0.7246	0.7523	0.8152
		XGBoost	0.8731	1.1903	8.2525	0.7011	0.7026	0.7823
		GRU	0.8658	1.3255	7.9467	0.6672	0.6745	0.7526
		ANN	0.9709	1.7241	9.0008	0.5671	0.5211	0.6354
	S2	DWT-KCPA-GWO-XGBoost	0.4933	0.4325	6.2351	0.8952	0.8623	0.9021
		KCPA-GWO-XGBoost	0.5352	0.4562	6.5232	0.8796	0.8325	0.8856
		GWO-XGBoost	0.5610	0.4795	6.6988	0.8766	0.7928	0.8632
		PSO-XGBoost	0.6631	0.7186	8.1075	0.8151	0.6945	0.7621
		XGBoos+ANN	0.6616	0.6729	0.6729	0.8268	0.7262	0.7553
		XGBoost	0.6323	0.5981	7.4515	0.8461	0.6729	0.7224
		GRU	0.7654	0.9101	8.3006	0.7658	0.6422	0.7026
		ANN	0.5725	0.5736	6.6371	0.8524	0.4934	0.5934
	S3	DWT-KCPA-GWO-XGBoost	0.2912	0.2001	4.0523	0.7823	0.8425	0.8463
		KCPA-GWO-XGBoost	0.3152	0.2245	4.6363	0.7652	0.8053	0.8215
		GWO-XGBoost	0.3990	0.2478	4.8954	0.7270	0.7713	0.8529
		PSO-XGBoost	0.5400	0.4193	6.5103	0.5384	0.6703	0.7421
		XGBoos-ANN	0.5223	0.4630	6.2914	0.4902	0.7026	0.7452
		XGBoost	0.4956	00.4059	6.0200	0.5531	0.6506	0.7116
		GRU	0.4321	0.3085	5.2589	0.6603	0.6295	0.7024
		ANN	0.4689	0.4552	5.5432	0.4988	0.4759	0.5663

### Further explanation

**Fig 12:**
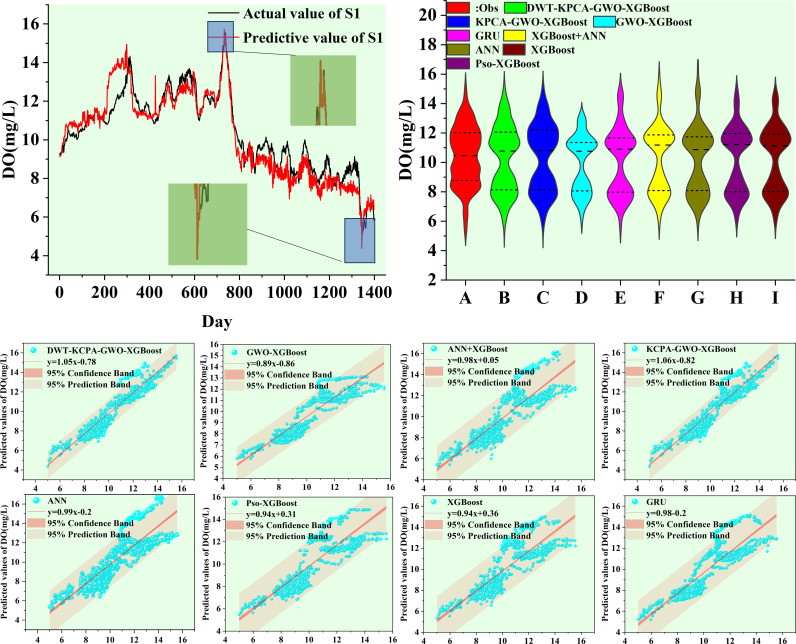
Predicted value of S1 station.

**Fig 13:**
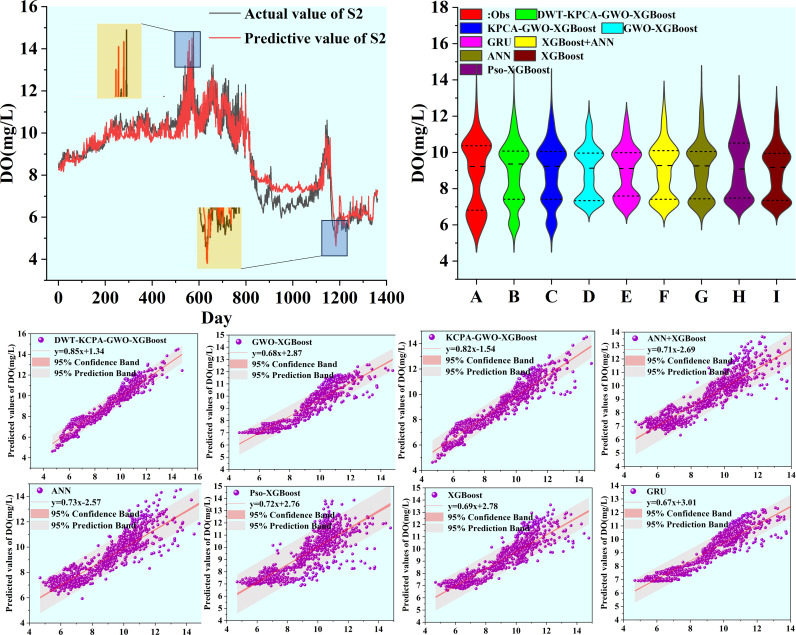
Predicted value of S2 station.

**Fig 14:**
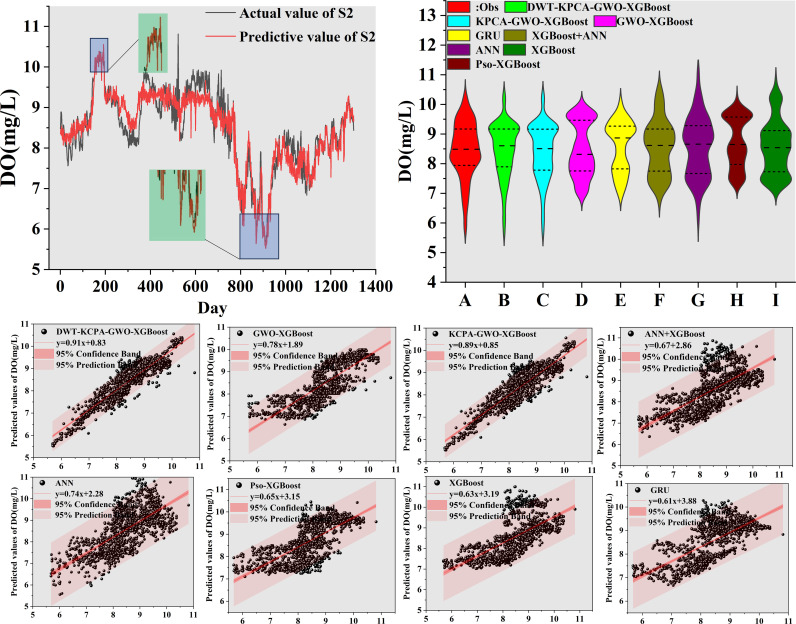
Predicted value of S3 station.

**Fig 15:**
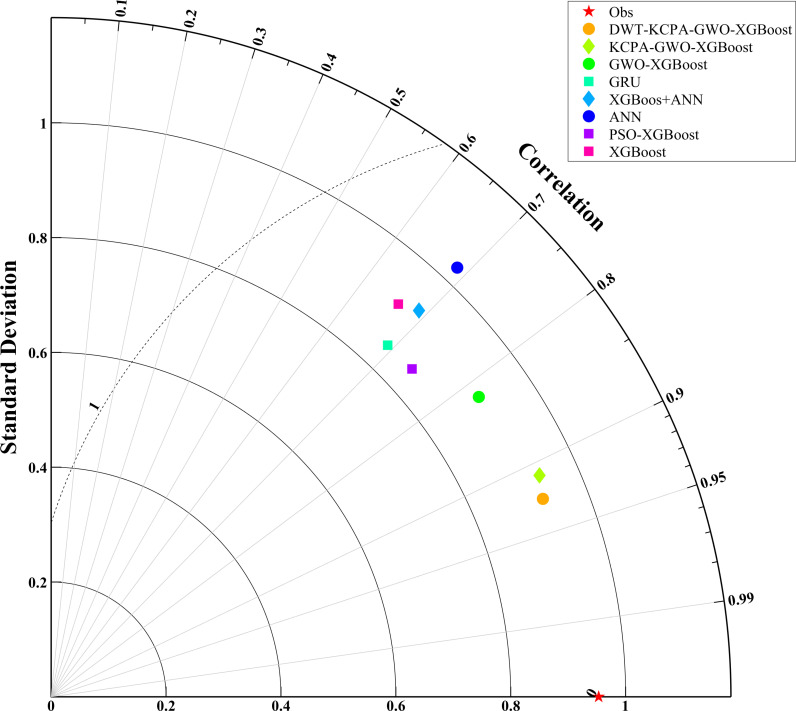
Performance comparison of eight models (S1).

**Fig 16:**
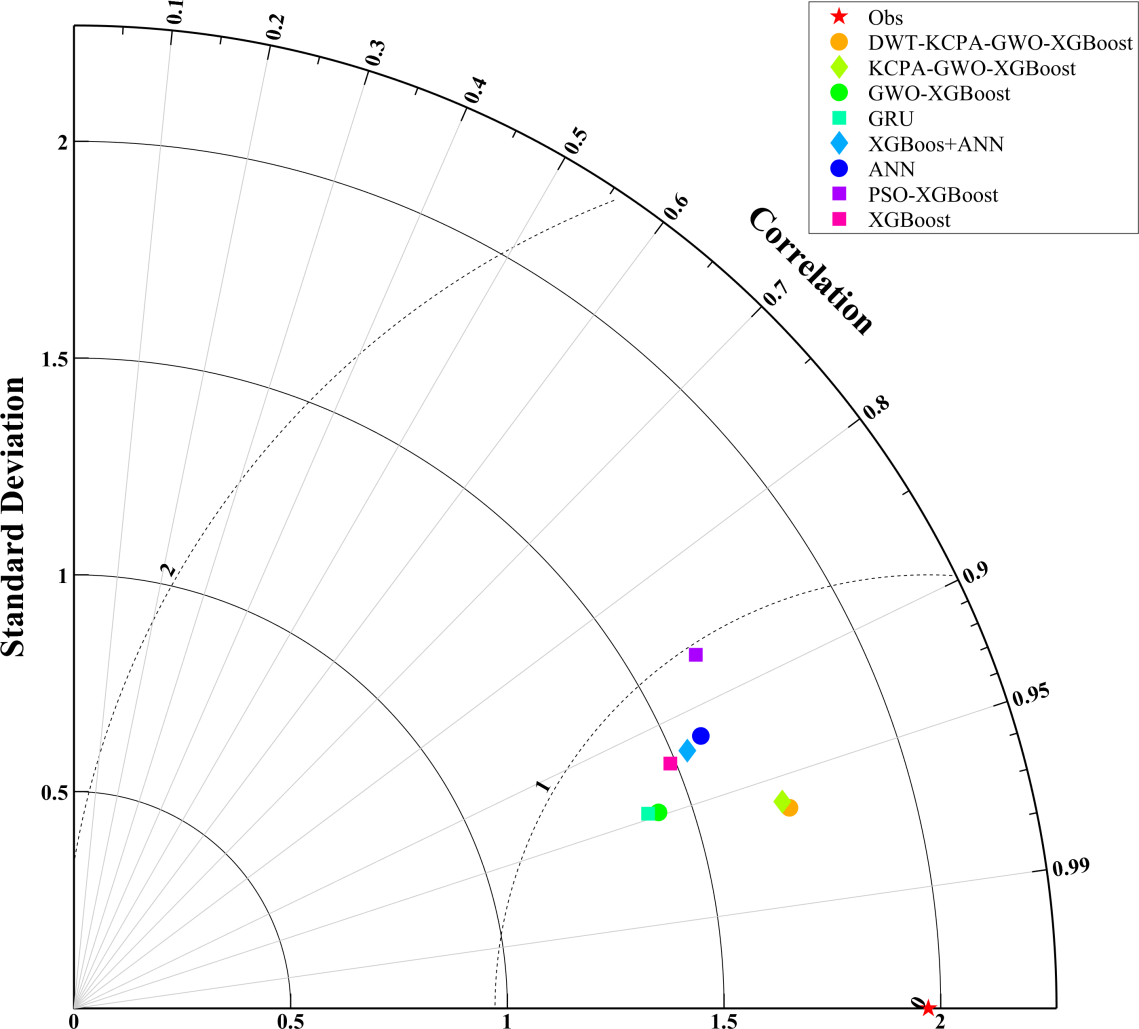
Performance comparison of eight models (S2).

**Fig 17:**
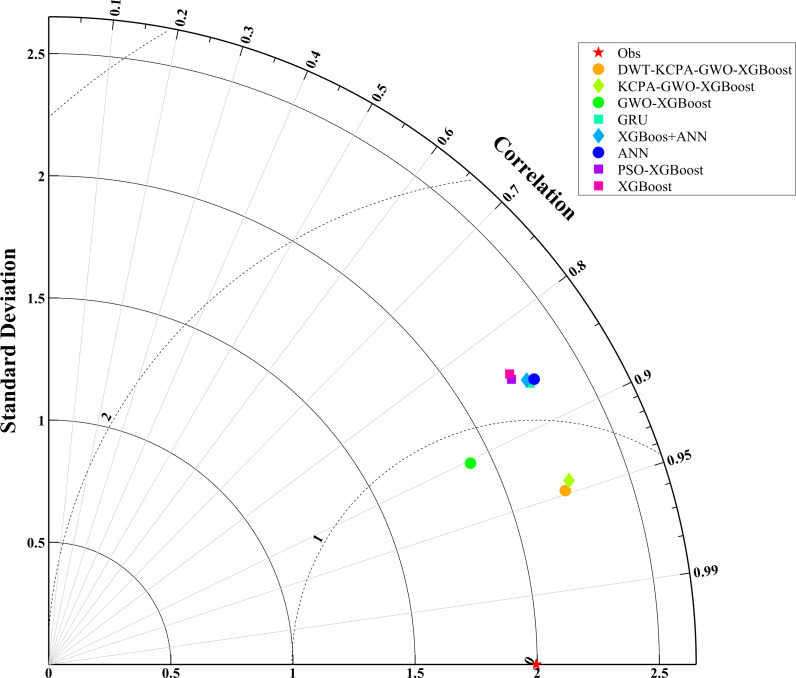
Performance comparison of eight models (S3).

We plotted scatter plots, violin plots and Taylor plots for the eight models at the three stations. The horizontal and vertical axes of the scatter plots represent the actual and predicted values of dissolved oxygen, respectively. The regression line equation indicates the fit of the scatterplot and the 95% confidence band is an assessment of the reliability of the model for the full data set. The 95% prediction band reflects the possible range of deviation for a single prediction. The violin plot provides information about the distribution of the data, which is divided into four parts by the dotted line representing the interquartile range of the data. As shown in Figs 12–14. To observe the performance of each model in a more intuitive way, we use a Taylor plot to represent the prediction results of each model. The arc represents the root mean square error, the line drawn from 0 represents the correlation coefficient, the ordinate represents the standard deviation and the abscissa represents the reference sequence. As shown in Figs 15–17.

### Peak prediction

Dissolved oxygen is an important indicator of the survival of aquatic organisms and the assessment of water quality. Excessive or insufficient dissolved oxygen can cause varying degrees of environmental damage. Excessive dissolved oxygen can accelerate eutrophication of the water body, while insufficient dissolved oxygen can inhibit the growth of aquatic organisms. The prediction of peaks is therefore an important standards for the evaluation of the model. We selected the maximum and minimum values from the test data and used our proposed model to predict the peak values at the three stations. The line graphs in Figs 12–14 show part of the model prediction sequence.

### Explore forecasted times

Predicting the future dissolved oxygen concentration in rivers plays a preventive and regulatory role in environmental monitoring, water quality management and ecological protection. We used DWT-KPCA-GWO-XGBoost to predict dissolved oxygen for the next 7, 15, 30 and 45 days and used several indicators to determine the best prediction time for the model. Prediction errors are shown in Table 4.

**Table 4:** Multi-step prediction error.

Model	Stations	Days	MAE	MAPE	MSE	NSE
DWT-KPCA-GWO-XGBoost	S1	7days	0.68	7.1	0.77	0.83
		15days	0.66	6.8	0.73	0.85
		30days	0.80	8.7	0.96	0.77
		45days	0.92	9.0	1.0	0.75
	S2	7days	0.58	6.8	0.53	0.85
		15days	0.55	6.0	0.49	0.87
		30days	0.71	8.2	0.68	0.79
		45days	0.98	10.5	0.95	0.69
	S3	7days	0.40	4.9	0.25	0.76
		15days	0.36	4.6	0.23	0.78
		30days	0.42	5.0	0.30	0.77
		45days	0.55	6.2	0.51	0.70

### Cross-validation and significance test

To comprehensively evaluate the performance of the dissolved oxygen prediction model and verify its generalization ability, we used the 5-fold cross-validation method. In this process, the dataset from the three stations was randomly divided into five equally-sized subsets (D1-D5). Each time, one of the subsets was selected as the test set, and the remaining four subsets were used as the training set. The model was trained and the NSE was calculated on the test set to evaluate the model performance. This process is repeated five times to ensure that each subset is used as a test set at least once, and the NSE value is used to measure the stability and generalization ability of the model on different subsets. In order to compare the performance difference between the DWT-KPCA-GWO-XGBoost model and other models, we performed a significance test on the NSE value in the cross-validation, that is, the DWT-KPCA-GWO-XGBoost model performed a sample t-test with the other seven models respectively. By calculating the t-value and p-value, we can systematically evaluate the significant differences between models [34]. The t-value reflects the degree of standardization of the difference between the NSE values of the two models, and the p-value is used to determine whether this difference is statistically significant. A p-value less than 0.05 indicates a significant difference between models, indicating that the two models are significantly different in terms of prediction performance. The results are shown in Table 5.

**Table 5:** Results of cross-validation and t-test.

stations	Models	NSE(D1)	NSE(D2)	NSE(D3)	NSE(D4)	NSE(D5)	t	p
S1	DWT-KCPA-GWO-XGBoost	0.8523	0.8621	0.8335	0.8245	0.8523	/	/
	KCPA-GWO-XGBoost	0.8142	0.8296	0.8011	0.7925	0.8235	3.36	0.009
	GWO-XGBoost	0.8155	0.8255	0.7952	0.7952	0.8150	3.88	0.004
	PSO-XGBoost	0.6826	0.7053	0.6925	0.6482	0.6882	13.7	<0.001
	XGBoos+ANN	0.7266	0.7056	0.6826	0.7042	0.7246	12.8	<0.001
	XGBoost	0.7003	0.6952	0.6625	0.7624	0.7011	7.9	<0.001
	GRU	0.6385	0.6156	0.6325	0.6651	0.6672	16.6	<0.001
	ANN	0.5265	0.5265	0.4926	0.5992	0.5671	15.3	<0.001
S2	DWT-KCPA-GWO-XGBoost	0.8934	0.8826	0.8525	0.8825	0.8952	/	/
	KCPA-GWO-XGBoost	0.8526	0.8516	0.8226	0.8426	0.8796	2.6	0.03
	GWO-XGBoost	0.8415	0.8425	0.8158	0.8352	0.8766	3.1	0.01
	PSO-XGBoost	0.7652	0.7925	0.7452	0.8223	0.8151	5.6	<0.001
	XGBoos+ANN	0.8203	0.8152	0.7456	0.8256	0.8268	4.3	0.02
	XGBoost	0.7956	0.8156	0.7559	0.8122	0.8461	4.5	0.01
	GRU	0.7256	0.7156	0.7452	0.7226	0.7658	12.2	<0.001
	ANN	0.6421	0.6652	0.5425	0.5225	0.5934	10.1	<0.001
S3	DWT-KCPA-GWO-XGBoost	0.7965	0.7856	0.7663	0.8153	0.7823	/	/
	KCPA-GWO-XGBoost	0.7263	0.7726	0.7456	0.8026	0.7652	2.4	0.04
	GWO-XGBoost	0.7253	0.7156	0.72566	0.6695	0.7266	5.6	<0.001
	PSO-XGBoost	0.5146	0.602	0.5036	0.6166	0.5384	9.6	<0.001
	XGBoos+ANN	0.5036	0.5123	0.4826	0.4252	0.4902	17.6	<0.001
	XGBoost	0.4526	0.3614	0.5153	0.5526	0.5531	8.1	<0.001
	GRU	0.6245	0.6035	0.6152	0.6715	0.6603	9.9	<0.001
	ANN	0.4826	0.3945	0.5052	0.5135	0.4988	9.9	<0.001

## Discussion

This study proposes a new method for a multi-data source and multi-algorithm fusion river dissolved oxygen prediction model. It aims to accurately predict non-linear dissolved oxygen time series data. The model parameters and prediction results are further explained below.

### Model hyperparameters

Given the limitations of traditional models, we combined different machine learning models to explore more efficient prediction methods. First, we believed that meteorological factors could drive the model to better capture the complex relationship of the dissolved oxygen sequence itself. However, considering that introducing too many meteorological factors would increase the complexity of the model, we used KPCA to reduce the dimensionality of the seven meteorological factors into four principal components. These four principal components contained more than 90% of the original sequence contained more than 90% of the original information. The core of KPCA is to use a kernel function to map the dissolved oxygen data from the original space into a high-dimensional feature space, and then to use traditional PCA to map the data into a low-dimensional space through an orthogonal transformation, so that the variance in the new space is maximised. In addition, we found that the feature matrix of the model has a lot of noise caused by conductivity and turbidity, so we use DWT to reduce the noise [35]. XGBoost is well suited to handle complex data tasks with multiple feature influences. However, the hyperparameter tuning has to be done manually or by traditional optimisation methods. To address this problem, we introduce the Grey Wolf optimiser into the model. The Grey Wolf Optimiser is a meta-heuristic algorithm that simulates the key behaviours of the four grey wolf species in nature, including prey seeking, prey surrounding and prey attacking. In combination with XGBoost, GWO explores and optimises the hyperparameter combination of XGBoost to find a set of high quality parameter settings. Measures such as introducing early stopping strategies and cross-validation, as well as controlling model complexity, are introduced to effectively prevent overfitting and improve the robustness and generalisability of the model. Table 6 shows the parameter settings used in this study. The KPCA algorithm of this study is run in MATLAB R22022a and GWO-XGBoost is run in python2022.

**Table 6:** Parameter settings.

GWO-XGBoost		
	objective	reg:squaregerror
	n_estimators	100
	maxdepth	1.79
	subsample	0.07
	test_size	0.2
	train_size	0.8
	random_state	42
	learning_rate	0.3
	min_child_weight	1
	Colsample_bytree	0.8
	gamma	0
	reg_lambda	1
	reg_alpha	0
	semrchAgents_no	3
	Max_iteration	50
KPCA		
	Target_dimension	4
	sigma	8
	Kernel function	RBF

This study used a 5-fold cross-validation method to evaluate the performance of eight dissolved oxygen prediction models, and selected NSE as the evaluation index. The results show that there are significant differences in the NSE values of different models on each data subset. As shown in Table 5, the DWT-KCPA-GWO-XGBoost model maintains a stable NSE in any subset of the test set. This shows that the model has strong generalization ability and can adapt to the prediction needs under different data distributions. In contrast, simple models such as ANN and GRU perform poorly, with the lowest NSE values, which indicates that they have poor generalization ability.

To further verify whether the performance differences between different models are statistically significant, we performed a two-by-two paired sample t-test on the five cross-validation results of each model [36]. As can be seen in Table 5, the p-value of the main model compared to the other models is less than 0.05, and the difference is statistically significant. It is not just a coincidence that the main model is more accurate.

### Results analysis

From the results in Table 3, the performance indicators of DWT-KPCA-GWO-XGBoost are better than KPCA-GWO-XGBoost, which shows that reducing the high-frequency noise of the model can improve the robustness of the prediction. When processing time series data with periodicity, DWT can help extract underlying, more periodic signals. Compared with the model using only GWO-XGBoost, the KPCA-GWO-XGBoost model fully considers the impact of meteorological changes on the water environment in dissolved oxygen prediction, showing stronger predictive ability. Therefore, meteorological factors such as temperature, humidity, air pressure and precipitation have a significant impact on the dissolved oxygen concentration in water bodies. Temperature is one of the main factors affecting dissolved oxygen concentration, because an increase in water temperature reduces the amount of oxygen that can be dissolved in the water, which directly affects the dissolved oxygen level in the water. In addition, meteorological factors such as precipitation and humidity also indirectly affect parameters such as river flow and water temperature, which in turn affect the amount of dissolved oxygen. For example, when precipitation increases, the flow rate and flow volume of the river may increase, which allows for better circulation and replenishment of dissolved oxygen in the water, and in turn affects the fluctuation of its concentration.

As can be seen in Fig 1, the historical record of dissolved oxygen shows seasonal changes. From January to July, there is a downward trend, and from July to December, there is an upward trend. The dissolved oxygen in the river is highest in winter and lowest in summer. The lowest values of dissolved oxygen at stations S1, S2 and S3 occurred on 2022/7/21, 2024/6/1 and 2021/8/19, with concentrations of 3.504766, 4.680931 and 1. 160716. The maximum values for dissolved oxygen at the three stations were 15.54437 on March 12, 2024, 17.02684 on February 10, 2023, and 13.12557 on December 25, 2021. This trend is closely related to the environment. Dissolved oxygen is closely related to factors such as climatic conditions, temperature, water flow and human activity in the watershed. Its seasonal changes are usually positively correlated with water temperature changes. As the temperature rises, the dissolved oxygen content in the water body will decrease, because a body of water with a high temperature can hold less oxygen. In addition, summer is the peak season for plant and aquatic life growth, and the decomposition rate of organic matter in the water accelerates, which further consumes dissolved oxygen. In winter, however, the low temperature inhibits the activity of aquatic life and the decomposition of organic matter, resulting in higher dissolved oxygen levels. In short, seasonal changes, climatic conditions and the ecological environment of the water body together determine the fluctuations in dissolved oxygen.

The scatter plot and violin plot together show the differences in performance and distribution characteristics of the different models in predicting dissolved oxygen. The DWT-KPCA-GWO-XGBoost model shows in the scatter plot that the data points are highly concentrated near the 45° diagonal line, indicating its high prediction accuracy and reliability. The violin plot further demonstrates that this model is closest to the observed value (Obs) in its ability to characterise the actual distribution and performs well in terms of distribution range, density and median. In contrast, models such as XGBoost and ANN show greater dispersion in the scatter plot, particularly in the high and low concentration areas where the deviation from the prediction is obvious. The violin plot also shows that these models have some degree of skew in the high concentration tail, which may be due to insufficient extreme value adaptivity or overfitting. Overall, the hybrid model significantly improves the distribution fitting ability through noise reduction and feature optimisation, and is suitable for predicting dissolved oxygen in complex environments.

There are many factors that affect the DO concentration and accurate prediction of the DO peaks is key to the model. In this study, we select six peaks from the test set data, which are the maximum or minimum values of the test set. From Figs 12–14, it can be seen that DWT-KPCA-GWO-XGBoost is able to predict the upper and lower limits of the sequences stably in the face of the different three data sets. Tables 7 and 8 show the prediction results for the six time periods.

**Table 7:** Minimum prediction results for the three stations.

Time	2024/6/21 20:00	2024/6/1 4:00:00	2024/4/25 0:00
Observed values	4.995695	4.680931	5.680158
predicted value	4.3668523	4.630489	5.6238605

**Table 8:** Maximum prediction results for the three stations.

Time	2024/3/12 16:00	2024/2/20 16:00	2024/2/20 16:00
Observed values	15.54437	14.78509	10.81486
predicted value	15.432719	12.439263	8.810918

In this study, dissolved oxygen data were additionally collected for the next 50 days to evaluate the prediction effectiveness of the proposed dissolved oxygen prediction model for different time periods in the future (7, 15, 30 and 45 days). By analysing the error metrics for different prediction periods, we found that the model was significantly better at predicting 15 days into the future than 7, 30 and 45 days.

Previous dissolved oxygen (DO) prediction models have generally failed to fully consider the impact of characteristic noise on model accuracy. This study, however, significantly improves the prediction accuracy of the model by denoising the input characteristics. Feature denoising not only reduces unnecessary noisy information, but also helps the model focus better on the most important DO change patterns, thereby optimizing the prediction results. In addition, we have also introduced meteorological factors into the DO prediction model, which have often been neglected in existing research. In this study, by incorporating meteorological factors into the model features, the model’s ability to adapt to environmental changes has been improved, thereby improving the accuracy of long-term predictions.

## Conclusion

In this study, a model incorporating multiple machine learning algorithms is proposed for predicting dissolved oxygen concentrations at three stations in the Yangtze River Basin. Facing the complexity of different hydrological conditions, the DWT-KPCA-GWO-XGBoost model shows a strong adaptive ability to effectively deal with the dynamic changes of hydrological variables, seasonal fluctuations, and external disturbances. By introducing Discrete Wavelet Transform (DWT) for feature extraction, the model is able to reduce the noise interference while maintaining the key information; meanwhile, Kernel Principal Component Analysis (KPCA) is employed to downscale the meteorological data into four main components, which are introduced into the prediction model. The prediction accuracy and generalization ability of the model were further improved by tuning XGBoost through the Gray Wolf Optimization (GWO) algorithm. The results show that the model not only outperforms the traditional methods in terms of prediction accuracy, but also excels in robustness, and is able to stably cope with the variations and uncertainties in different hydrological environments, demonstrating strong practicality and application potential.

In this study, the prediction of peak dissolved oxygen concentration and the optimal prediction time are discussed in depth. The experimental results verified the accuracy and validity of the proposed model. In order to demonstrate more intuitively the differences in prediction accuracy between models and their matching with measured data, Taylor diagrams and violin plots are used in this study to clearly compare the prediction performance of different models and to reveal the consistency and differences in the structural distribution of measured and predicted data. Based on our experimental results, it can be proved that the introduction of meteorological elements and noise reduction processing play an important role in improving the model prediction accuracy. Meteorological elements as external drivers provide key information for the model, while the noise reduction method further optimizes the prediction performance by removing the noise from the model features.

In this study, the performance of eight dissolved oxygen prediction models was systematically evaluated by 5-fold cross-validation and paired-sample t-test. The results showed that our proposed DWT-KCPA-GWO-XGBoost model exhibited the best prediction performance with better NSE values than the other models on all the tested subsets. In addition, the t-test results show that the performance difference between the DWT-KCPA-GWO-XGBoost model and the other seven models is statistically significant, a result that suggests that the prediction accuracy of the model can be effectively improved by feature noise reduction and the introduction and optimization of meteorological factors. The importance of feature extraction and driving variable selection for DO prediction model construction was further verified.

Despite the relatively good research results, further research is needed to comprehensively assess water quality in the aquatic environment. Water quality assessment is not only based on dissolved oxygen concentration, but also needs to consider other key water quality parameters, such as pH, phosphorus, nitrogen, turbidity, chlorophyll, potassium permanganate index, ammonia nitrogen, etc., which together affect the ecological health and water quality of the water body. Therefore, future research should expand the scope of prediction and integrate multiple water quality parameters.

Although we have clearly demonstrated that meteorological factors can influence model predictions, we have not confirmed which specific meteorological factors have a significant impact on the predictive effectiveness of the model. Future research could further investigate and analyse how different meteorological parameters, such as temperature, humidity, precipitation, wind speed, etc., individually or jointly contribute to changes in dissolved oxygen concentration. In addition, the different effects of different meteorological factors in different seasons or weather conditions can be considered, thus providing more basis for model optimisation. By analysing meteorological factors in more detail, we can better understand the driving mechanisms of water quality changes.

Finally, we plan to cooperate with government agencies and environmental protection organisations to further promote the integration of the proposed model into the water environment monitoring system. This cooperation will not only help strengthen the scientific management and protection of the water environment, but also provide more accurate technical support for water quality monitoring and ecological protection. By applying the model to real-world environmental monitoring, it can provide water resource managers with quantitative analytical tools to help identify and predict potential water quality problems and take timely and effective action to prevent water pollution and ecological degradation. In addition, the model proposed in this study has strong generalisability, which is not only of practical value for water quality management in the Yangtze River Basin, but can also be extended to water environmental protection in other regions, thus contributing to the sustainable management of water resources globally.

## References

[1] Hutchins MG, Qu Y, Charlton MB. Successful modelling of river dissolved oxygen dynamics requires knowledge of stream channel environments. J Hydrol. 2021;603:126991. doi: 10.1016/j.jhydrol.2021.126991

[2] Xu C, Luo P, Wu P, Song C, Chen X. Detection of periodicity, aperiodicity, and corresponding driving factors of river dissolved oxygen based on high-frequency measurements. J Hydrol. 2022;609:127711. doi: 10.1016/j.jhydrol.2022.127711

[3] Zehra R, Singh SP, Verma J, Kulshreshtha A. Spatio-temporal investigation of physico-chemical water quality parameters based on comparative assessment of QUAL 2Kw and WASP model for the upper reaches of Yamuna River stretching from Paonta Sahib, Sirmaur district to Cullackpur, North Delhi districts of North India. Environ Monit Assess. 2023;195(4):480. doi: 10.1007/s10661-023-11072-5 36930328

[4] Zhang X. Simulation study on the impact of South–North water transfer central line recharge on the water environment of Bai River. Water. 2023;15(10):1871.

[5] Li D, Sun Y, Sun J, Wang X, Zhang X. An advanced approach for the precise prediction of water quality using a discrete hidden Markov model. J Hydrol. 2022;609:127659. doi: 10.1016/j.jhydrol.2022.127659

[6] Huang S, Wang Y, Xia J. Which riverine water quality parameters can be predicted by meteorologically-driven deep learning? Sci Total Environ. 2024;946:174357. doi: 10.1016/j.scitotenv.2024.174357 38945234

[7] Li S, Qasem SN, Band SS, Ameri R, Pai H-T, Mehdizadeh S. Explainable machine learning models for estimating daily dissolved oxygen concentration of the Tualatin River. Eng Appl Comput Fluid Mech. 2024;18(1):2304094. doi: 10.1080/19942060.2024.2304094

[8] Wang Z, Wang Q, Liu Z, Wu T. A deep learning interpretable model for river dissolved oxygen multi-step and interval prediction based on multi-source data fusion. J Hydrol. 2024;629:130637. doi: 10.1016/j.jhydrol.2024.130637

[9] Singh RB, Patra KC, Pradhan B, Samantra A. HDTO-DeepAR: a novel hybrid approach to forecast surface water quality indicators. J Environ Manage. 2024;352:120091. doi: 10.1016/j.jenvman.2024.120091 38228048

[10] Tian Y, Zhao Y, Yin Z, Deng N, Li S, Zhao H, et al. Integrating spatial-temporal features into prediction tasks: A novel method for identifying the potential water pollution area in large river basins. J Environ Manage. 2025;373:123522. doi: 10.1016/j.jenvman.2024.123522 39632310

[11] Zhu S, Heddam S. Prediction of dissolved oxygen in urban rivers at the Three Gorges Reservoir, China: extreme learning machines (ELM) versus artificial neural network (ANN). Water Qual Res J. 2019;55(1):106–18. doi: 10.2166/wqrj.2019.053

[12] Achite M, Katipoğlu OM, Elshaboury N, Kartal V, Aktürk G, Ertugay N. Modeling of irrigation water quality parameter (sodium adsorption ratio) using hybrid swarm intelligence-based neural networks in a semi-arid environment at SMBA dam, Algeria. Theor Appl Climatol. 2024;155(8):8299–318. doi: 10.1007/s00704-024-05109-z

[13] Su T, Shi Y, Yu J, Yue C, Zhou F. Nonlinear compensation algorithm for multidimensional temporal data: a missing value imputation for the power grid applications. Knowl Based Syst. 2021;215:106743. doi: 10.1016/j.knosys.2021.106743

[14] Chen T, Guestrin C. Xgboost: A scalable tree boosting system. Proceedings of the 22nd ACM Sigkdd International Conference on Knowledge Discovery and Data Mining. 2016.

[15] Zhang H, Si S, Hsieh C-J. GPU-acceleration for large-scale tree boosting. arXiv preprint. 2017. doi: arXiv:1706.08359

[16] Chen T, et al. Xgboost: extreme gradient boosting. R package version 0.4-2 1. 2015;4:1–4.

[17] Mitchell R, Frank E. Accelerating the XGBoost algorithm using GPU computing. PeerJ Comput Sci. 2017;3:e127. doi: 10.7717/peerj-cs.127

[18] Samat A, Li E, Wang W, Liu S, Lin C, Abuduwaili J. Meta-XGBoost for hyperspectral image classification using extended mser-guided morphological profiles. Remote Sensing. 2020;12(12):1973. doi: 10.3390/rs12121973

[19] Mirjalili S, Mirjalili SM, Lewis A. Grey wolf optimizer. Adv Eng Softw. 2014;69:46–61.

[20] Zhou J, Qiu Y, Zhu S, Armaghani DJ, Li C, Nguyen H, et al. Optimization of support vector machine through the use of metaheuristic algorithms in forecasting TBM advance rate. Eng Appl Artif Intell. 2021;97:104015. doi: 10.1016/j.engappai.2020.104015

[21] Srivastava VK, Prasad D. DWT-based feature extraction from ECG signal. Am J Eng Res (AJER). 2013;2(3):44–50.

[22] Meng X, Bao Y, Ye Q, Liu H, Zhang X, Tang H, et al. Soil organic matter prediction model with satellite hyperspectral image based on optimized denoising method. Remote Sens. 2021;13(12):2273. doi: 10.3390/rs13122273

[23] Uddin MG, Nash S, Mahammad Diganta MT, Rahman A, Olbert AI. Robust machine learning algorithms for predicting coastal water quality index. J Environ Manage. 2022;321:115923. doi: 10.1016/j.jenvman.2022.115923 35988401

[24] Uddin MG, Nash S, Rahman A, Olbert AI. A novel approach for estimating and predicting uncertainty in water quality index model using machine learning approaches. Water Res. 2023;229:119422. doi: 10.1016/j.watres.2022.119422 36459893

[25] Uddin MG, Nash S, Rahman A, Olbert AI. Assessing optimization techniques for improving water quality model. J Clean Prod. 2023;385:135671. doi: 10.1016/j.jclepro.2022.135671

[26] Sharif O, Hasan MZ, Rahman A. Determining an effective short term COVID-19 prediction model in ASEAN countries. Sci Rep. 2022;12(1):5083. doi: 10.1038/s41598-022-08486-5 35332192 PMC8943510

[27] Park S, Park S, Hwang E. Normalized residue analysis for deep learning based probabilistic forecasting of photovoltaic generations. In: 2020 IEEE International Conference on Big Data and Smart Computing. BigComp, IEEE; 2020. p. 483–486.

[28] Arora P, Jalali SMJ, Ahmadian S, Panigrahi BK, Suganthan PN, Khosravi A. Probabilistic wind power forecasting using optimized deep auto-regressive recurrent neural networks. IEEE Trans Ind Inf. 2023;19(3):2814–25. doi: 10.1109/tii.2022.3160696

[29] Jin T, Cai S, Jiang D, Liu J. A data-driven model for real-time water quality prediction and early warning by an integration method. Environ Sci Pollut Res Int. 2019;26(29):30374–85. doi: 10.1007/s11356-019-06049-2 31440975

[30] Katipoğlu OM. Combining discrete wavelet decomposition with soft computing techniques to predict monthly evapotranspiration in semi-arid Hakkâri province, Türkiye. Environ Sci Pollut Res Int. 2023;30(15):44043–66. doi: 10.1007/s11356-023-25369-y 36680720

[31] Yang Y, Tu J, Shen W. kCPA: Towards sensitive pointer full life cycle authentication for OS kernels. IEEE T Depend Secure Comput. 2023;31.

[32] Mohammed H, Tornyeviadzi HM, Seidu R. Modelling the impact of weather parameters on the microbial quality of water in distribution systems. J Environ Manage. 2021;284:111997. doi: 10.1016/j.jenvman.2021.111997 33524868

[33] He M, Wu S, Huang B, Kang C, Gui F. Prediction of total nitrogen and phosphorus in surface water by deep learning methods based on multi-scale feature extraction. Water. 2022;14(10):1643. doi: 10.3390/w14101643

[34] Opejin A, Park YM. Assessing bias in personal exposure estimates when indoor air quality is ignored: a comparison between GPS-enabled mobile air sensor data and stationary outdoor sensor data. Sci Total Environ. 2024;950:175249. doi: 10.1016/j.scitotenv.2024.175249 39098424

[35] Achite M, Katipoglu OM, Şenocak S, Elshaboury N, Bazrafshan O, Dalkılıç HY. Modeling of meteorological, agricultural, and hydrological droughts in semi-arid environments with various machine learning and discrete wavelet transform. Theor Appl Climatol. 2023;154(1–2):413–51. doi: 10.1007/s00704-023-04564-4

[36] Song S, Chen X, Hu Z, Zan C, Liu T, De Maeyer P, et al. Deciphering the impact of wind erosion on ecosystem services: an integrated framework for assessment and spatiotemporal analysis in arid regions. Ecol Indic. 2023;154:110693. doi: 10.1016/j.ecolind.2023.110693

